# Martian CO_2_ Ice Observation at High Spectral Resolution With ExoMars/TGO NOMAD

**DOI:** 10.1029/2021JE007083

**Published:** 2022-05-04

**Authors:** F. Oliva, E. D’Aversa, G. Bellucci, F. G. Carrozzo, L. Ruiz Lozano, F. Altieri, I. R. Thomas, O. Karatekin, G. Cruz Mermy, F. Schmidt, S. Robert, A. C. Vandaele, F. Daerden, B. Ristic, M. R. Patel, J.‐J. López‐Moreno, G. Sindoni

**Affiliations:** ^1^ Istituto di Astrofisica e Planetologia Spaziali (IAPS/INAF) Rome Italy; ^2^ Université Catholique de Louvain‐la‐Neuve (UCLouvain) Louvain‐la‐Neuve Belgium; ^3^ Royal Observatory of Belgium Brussels Belgium; ^4^ Royal Belgian Institute for Space Aeronomy (IASB‐BIRA) Brussels Belgium; ^5^ CNRS GEOPS Université Paris‐Saclay Orsay France; ^6^ Institut Universitaire de France (IUF) Paris France; ^7^ School of Physical Sciences The Open University Milton Keynes UK; ^8^ Instituto de Astrofìsica de Andalucia (IAA) Consejo Superior de Investigaciones Científicas (CSIC) Granada Spain; ^9^ Agenzia Spaziale Italiana (ASI) Rome Italy

**Keywords:** Mars, Mars surface, surface properties, surface ice, CO_2_ ice, CO_2_ ice clouds

## Abstract

The Nadir and Occultation for MArs Discovery (NOMAD) instrument suite aboard ExoMars/Trace Gas Orbiter spacecraft is mainly conceived for the study of minor atmospheric species, but it also offers the opportunity to investigate surface composition and aerosols properties. We investigate the information content of the Limb, Nadir, and Occultation (LNO) infrared channel of NOMAD and demonstrate how spectral orders 169, 189, and 190 can be exploited to detect surface CO_2_ ice. We study the strong CO_2_ ice absorption band at 2.7 μm and the shallower band at 2.35 μm taking advantage of observations across Martian Years 34 and 35 (March 2018 to February 2020), straddling a global dust storm. We obtain latitudinal‐seasonal maps for CO_2_ ice in both polar regions, in overall agreement with predictions by a general climate model and with the Mars Express/OMEGA spectrometer Martian Years 27 and 28 observations. We find that the narrow 2.35 μm absorption band, spectrally well covered by LNO order 189, offers the most promising potential for the retrieval of CO_2_ ice microphysical properties. Occurrences of CO_2_ ice spectra are also detected at low latitudes and we discuss about their interpretation as daytime high altitude CO_2_ ice clouds as opposed to surface frost. We find that the clouds hypothesis is preferable on the basis of surface temperature, local time and grain size considerations, resulting in the first detection of CO_2_ ice clouds through the study of this spectral range. Through radiative transfer considerations on these detections we find that the 2.35 μm absorption feature of CO_2_ ice clouds is possibly sensitive to nm‐sized ice grains.

## Key Points

- Martian surface CO_2_ ice detection at high spectral resolution with Trace Gas Orbiter/Nadir and Occultation for MArs Discovery
- General good agreement of the seasonal surface CO_2_ ice maps with Mars Express/OMEGA observations and with Mars Climate Database predictions
- CO_2_ ice clouds detection through the 2.35 micron CO_2_ ice absorption band, likely sensitive to a population of nm‐sized ice grains

## 1 Introduction

CO_2_ is the main component of the Martian atmosphere, in which temperatures can be low enough to induce local condensation of the molecule as ice/snow on the surface or as clouds at high altitude (Aoki et al., 2018; Clancy & Sandor, 1998; Clancy et al., 2007, 2019; Langevin et al., 2007; McConnochie et al., 2010; Montmessin et al., 2007; Schmidt et al., 2009, 2010; Schofield et al., 1997; Vincendon et al., 2011). The presence of CO_2_ ice on the surface has been widely investigated remotely in different spectral ranges by instruments aboard several missions, such as Mariner 9/IRIS (Hanel et al., 1972), Viking/IRTM (Kieffer et al., 1972), Mars Global Surveyor/TES (Christensen et al., 2001), Mars Express OMEGA (Bibring et al., 2004) and PFS (Formisano et al., 2005) spectrometers, and the Mars Reconnaissance Orbiter/CRISM instrument (Murchie et al., 2007). CO_2_ ice is mainly found at high latitude in form of seasonal cap (Andrieu et al., 2018; Brown et al., 2010, 2012; Herr & Pimentel, 1969; Kieffer & Titus, 2001; Langevin et al., 2007; Larson & Fink, 1972; Schmidt et al., 2009, 2010) and its physical and spatial characteristics strongly affect (and are affected by) the climatology of its gaseous counterpart (Forget, 1998; Forget et al., 1995; Leighton & Murray, 1966). Models and surface pressure measurements indicate that more than one quarter of atmospheric CO_2_ deposits seasonally on each hemisphere (Forget et al., 1998; Kieffer & Titus, 2001; Tillman et al., 1993). On the other hand, the freezing of atmospheric CO_2_ over the polar caps has an impact on the total atmospheric pressure of the planet (e.g., Leighton & Murray, 1966). In general, the sublimation/condensation processes of CO_2_ on Mars have a fundamental role in sculpting the planet's energy budget (e.g., Kieffer, 1979).

Spectrally wide CO_2_ ice absorption bands can be investigated in the infrared spectral range between 1 and 5 μm (Larson & Fink, 1972; Schmitt et al., 1998). This interval is commonly covered by multispectral imaging spectrometers, such as Mars Express/OMEGA and Mars Reconnaissance Orbiter/CRISM, operating at a moderate spectral resolution (resolving power ∼10^2^). Studies of surface ice at higher spectral resolution (resolving power ∼10^3^) have been performed from ground‐based telescopes allowing differentiation of the polar caps' CO_2_ frost spectral features from those related to gaseous CO_2_ (Larson & Fink, 1972). The same resolution has been reached by the Mars Express/PFS instrument studying the northern seasonal cap (Giuranna et al., 2007). On the other hand, an analysis with even higher spectral resolution (resolving power ∼10^4^) from space, has not yet been performed.

ExoMars Trace Gas Orbiter (TGO)/Nadir and Occultation for MArs Discovery (NOMAD) is a suite of three spectrometers conceived to study the Martian atmosphere at high spectral resolution (Section 2). The first analyses of its data were devoted to methane, other trace gases, and suspended aerosols abundances and climatology (Aoki et al., 2019; Gérard et al., 2020; Korablev et al., 2019; Liuzzi et al., 2019, 2020; López‐Valverde et al., 2018; Vandaele et al., 2019, to cite a few). In this work we start exploring the NOMAD data set information content in order to assess the feasibility of surface ices studies.

Since the instrumental characteristics are not optimal for surface spectroscopy, we adopt a semi‐qualitative approach aimed to assess the sensitivity of the data set to the presence of CO_2_ ice exposed on the surface and its discrimination from surface water ice and clouds. Moreover, we discuss the potential of the CO_2_ ice 2.35 μm absorption band for the retrieval of the ice grains size and optical depth. This kind of investigation represents a useful framework for more quantitative and extended retrievals of CO_2_ ice properties, which will be discussed in follow‐up papers (Section 6). We include data spanning the second half of Martian Year (MY) 34 and the first half of MY35. In particular, MY34 data cover the 2018 global dust storm (Guzewich et al., 2019; Kass et al., 2019; Smith & Guzewich, 2019; Viúdez‐Moreiras et al., 2019), allowing considerations on how suspended dust can affect the ice identification.

The tools and the methodology of the analysis are described in Section 3 and 4 respectively and the results are debated in Section 5. Finally, our conclusions are given in Section 6 along with the discussion about possible future work on this topic.

## 2 Instrument and Data Processing

### 2.1 The NOMAD Spectrometer

The NOMAD instrument is a spectrometer suite aboard the ESA/Roscosmos ExoMars TGO spacecraft. NOMAD is capable of studying both the atmosphere and the surface of Mars, with primary focus on the investigation of trace gases. It observes with different pointing geometries taking advantage of three channels: UVIS, operating in the ultraviolet/visible range 0.2–0.65 μm, working both in nadir and solar occultation geometries; Limb, Nadir, and Occultation (LNO), covering the infrared range 2.2–3.8 μm, working in nadir, limb and solar occultation geometries; SO, working in the range 2.3–4.3 μm and performing dedicated solar occultation measurements (Neefs et al., 2015). While UVIS design is inherited from the ultraviolet/visible spectrometer developed for the ExoMars lander mission as part of the Humboldt payload (Patel et al., 2017; Vandaele et al., 2015), both LNO and SO channels share the same design of the echelle grating Solar Occultation in the InfraRed (SOIR) spectrometer, part of the SPICAV instrument on board the Venus Express (VEx) spacecraft (Nevejans et al., 2006). They do not acquire the data in the whole spectral range at once, but only in small portions, in spectral regions of approximately 22 cm^−1^, determined by the bandpass (hereafter called “order”) selected on a tunable entrance filter (Acousto‐Optical‐Tunable‐Filter, AOTF, Section 2.2). Both LNO and UVIS perform nadir observations and, hence, can be used to study the surface of Mars. In this work we focus on the investigation of LNO information content alone, while UVIS investigation will be covered in a follow‐up paper in preparation. For a complete explanation of the LNO instrumental structure and operation the reader is referred to Neefs et al. (2015) and Thomas et al. (2016). In the next section we report the main issues of the LNO operations that strongly affect the processes of calibration and modeling of the measurements within the framework of our investigation. The main instrumental characteristics of LNO for the nadir geometry are reported in Table 1.

**Table 1:** Limb, Nadir, and Occultation (LNO) Channel Instrumental Characteristics for Nadir Geometry (Liuzzi et al., 2019; Neefs et al., 2015; Vandaele et al., 2018)

LNO Channel Instrumental Characteristics (NADIR)	
Wavelength range *λ*	2.3–3.8 (μm)
Wavenumber range *k*	2630–4,250 (cm^−1^)
Resolving power *λ*/Δ*λ*	10^4^
Field of view	4 × 150 (arcmin^2^)
Instantaneous footprint (400 km orbit)	0.5 × 17.5 (km^2^)
Average integration time	15 (s)
Signal‐to‐noise ratio	100

### 2.2 Data Calibration and Uncertainties

The shape of the raw NOMAD‐LNO spectra is strongly modulated by the AOTF spectral transmission curve and by the spectral response of the grating (the so‐called Blaze function). Both curves are characterized by strong peaks, whose exact position on the detector is determined differently and changes from one order to the other. The AOTF peak position is tunable through an internal radio frequency generator, whereas the peak width, which defines the nominal spectral range assigned to a given order, is nearly constant (∼22 cm^−1^). On the other hand, while the Blaze function is well approximated by a Gaussian curve, the AOTF transmission curve includes several secondary peaks (side‐lobes, i.e., relative maxima at both sides of the main lobe) and is better modeled as a combination of a sinus‐cardinal with a Gaussian (Liuzzi et al., 2019). It is worth noting that the presence of the side‐lobes in the AOTF curve allows photons from a spectral range much wider than the nominal to fall on the grating. The signal produced by these unwanted photons sums up with the nominal one, in different locations on the detector, yielding a partial mixing of spectral information, more and more significant in moving from the center to the edges of any order. As a result, LNO spectra can show misplaced spectral signatures, that is, features pertaining to a given wavelength placed or replicated in a wrong position. We have to point out that the conversion from raw to radiance spectra implies the full correction of the lobed shape typical of raw spectra. However, even if the instrumental parameters of relevance were known with high accuracy (and this is not always the case), the partial spectral mixing due to the side‐lobes cannot be removed by the radiometric calibration procedure.

The complete method for converting raw data to reflectance factor is given in Thomas et al. (2021) while an alternative equivalent method is given in Cruz Mermy et al. (2021). In short, occasional calibration measurements are made where the LNO channel directly observes the Sun—albeit with a much shorter integration time—which are then used as a known reference source to calibrate the Mars nadir spectra. Corrections are applied to account for differences in instrument temperature between the solar spectra and the Mars spectra, and the spectral calibration is corrected by fitting to strong solar and/or atmospheric absorption lines.

Once the solar and nadir spectra have been normalized to counts per pixel per second, the reflectance factor conversion is then:

(1) $$R=\left(C_{nadir}{d_{Mars}}^{2}\right)\;/\;\left(C_{solar}{r_{Sun}}^{2}\mu_{0}\right)$$

where *C*_nadir_ and *C*_solar_ are the LNO measured nadir and solar counts, *d*_Mars_ is the Sun‐Mars distance, *r*_Sun_ is the solar radius and *µ*_0_ = cos(SZA), SZA being the Solar Zenith Angle.

The spectral mixing issue due to the AOTF side‐lobes also propagates to the reflectance calibration. In this case, this effect also introduces low‐frequency oscillations in the spectral continuum, making the direct comparison in absolute values between measurements and radiative transfer models complex. For the above reasons, we only deal with the NOMAD intensities close to the center of each spectral order, being less affected by the aforementioned issues, or with relative quantities obtained through normalization and continuum‐removal processing.

The main source of noise in LNO observations comes from the thermal background of the instrument itself (Thomas et al., 2016). The channel operates at around −10 to +10°C, and so emission at infrared wavelengths reaches the detector, contributing to the majority of the signal recorded by the detector. On board background subtraction is employed to remove this contribution from the spectra, by taking consecutive Mars and dark frames (where the AOTF is switched off) and subtracting the dark frames from the Mars frames to leave the Mars signal which is then transmitted to Earth. As the thermal background is the principal source of radiation, the integration time is limited by the temperature of the instrument ‐ and as saturation of the detector must always be avoided, a conservative value of around 200 ms is used. However, a shorter integration time means that more frames can be co‐added on board, up to a maximum period defined by the channel's measurement cycle (Thomas et al., 2016). For standard nadir observations, this period is calculated by dividing 15 s by the number of diffraction orders measured (LNO typically measures 2, 3, or 4 diffraction orders per observation).

### 2.3 Data Selection

**Figure 1:**
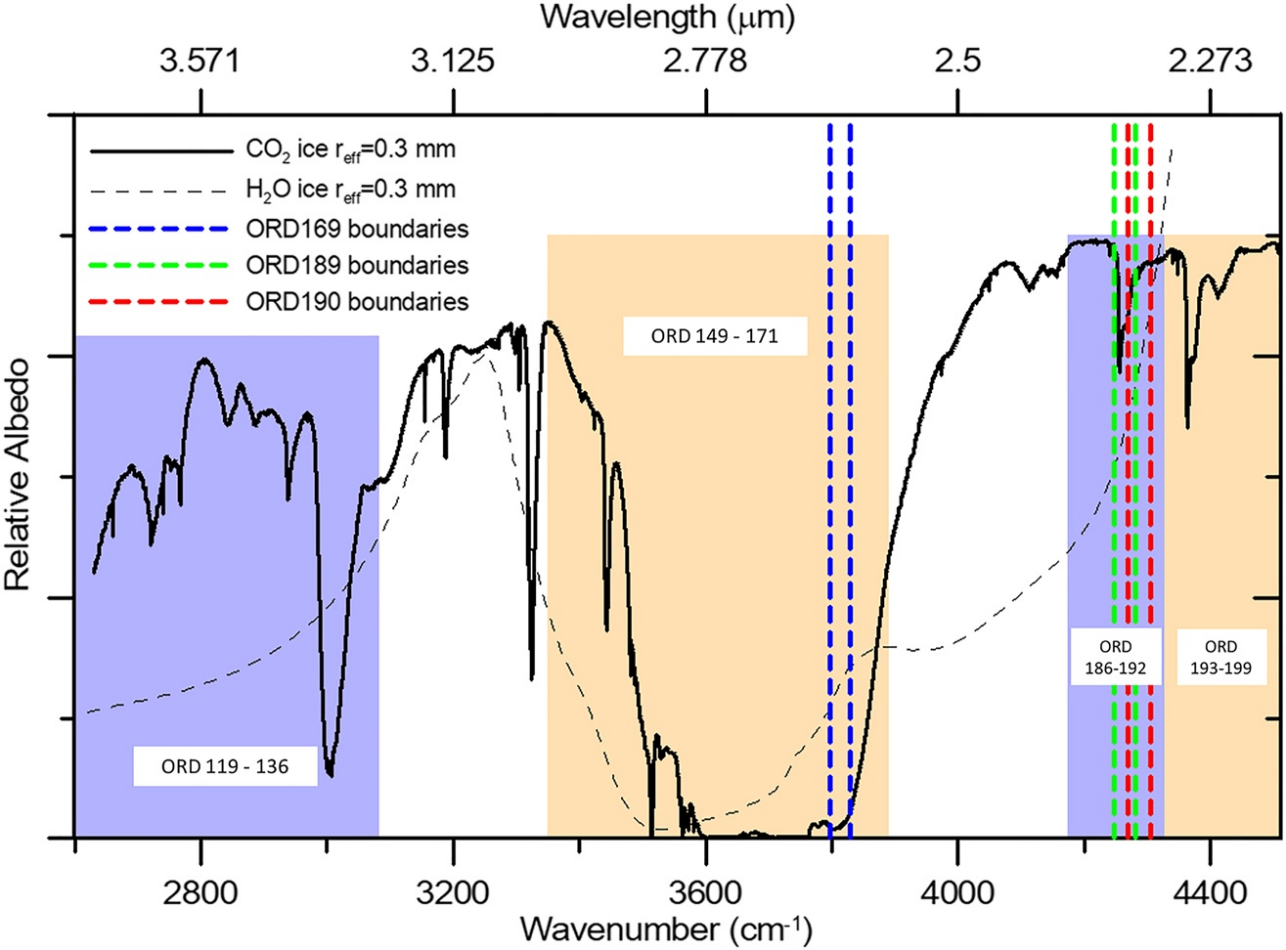
CO_2_ ice (thick solid black line) and H_2_O ice (thin dashed black line) albedo spectra (see Section 3.1 for surface ice albedo modeling details). The ranges covered by Limb, Nadir, and Occultation orders described in Section 2.3 (Table 2) are highlighted in colored shaded rectangles. Orders 169, 189, and 190 ranges are highlighted by the blue, green and red vertical dashed lines respectively.

Several CO_2_ ice spectral features fall within the LNO spectral range and are covered by different diffraction orders (Figure 1; Table 2). Orders 193 through 199, conceived for dust and surface nadir observations, cover the range 2.22–2.31 μm. In particular, orders 193 and 194, combined together, cover the 2.28 μm CO_2_ ice absorption band. Orders 186 through 192, nominally devoted to the study of atmospheric CO, cover the spectral range 2.30–2.39 μm. This interval contains the 2.35 μm CO_2_ ice absorption band that is well covered by order 189. Orders 149 through 171, dedicated to the study of gaseous CO_2_ and H_2_O, cover the strong 2.7 μm CO_2_ and H_2_O ices absorption bands and several weaker CO_2_ ice bands within the 2.58–2.98 μm range. Finally, orders 119 through 136, dedicated to atmospheric HDO, CO_2_, H_2_O, and CH_4_, cover different moderate to weak CO_2_ ice absorption bands within the range 3.25–3.74 μm.

**Table 2:** Diffraction Orders Covering CO_2_ Ice Spectral Features Within Limb, Nadir, and Occultation Spectral Range

Order	Wavelength range (μm)	Wavenumber range (cm^−1^)	Main focus
119–136	3.245–3.738	2674.90–3081.44	HDO/CO_2_/CH_4_/H_2_O (gas)
149–171	2.581–2.986	3349.24–3874.46	H_2_O/CO_2_ (gas)
186–192	2.299–2.392	4180.93–4440.90	CO (gas)
193–199	2.218–2.305	4338.28–4508.88	Nadir dust/surface
**169**	**2.612–2.632**	**3798.80–3829.15**	**H**_**2**_ **O (gas)**
**189**	**2.335–2.354**	**4248.36–4282.30**	**CO (gas)**
**190**	**2.323–2.341**	**4270.84–4304.96**	**CO (gas)**

*Note.* Orders in bold are the ones selected for the analysis described in Section 4.

These orders are not all equally effective for our purpose, that is to establish the CO_2_ ice detection capability of NOMAD data, from the point of view of both spectral and spatial coverage. Among all the aforementioned CO_2_ ice features, the 2.7 μm absorption band is the strongest in the LNO range, hence the most sensitive to small ice abundances, but it has the side effect of easily becoming saturated, especially on Martian ice‐abundant deposits where large slabs (>20 cm) are expected (Andrieu et al., 2018; Langevin et al., 2007). Moreover, it is worth stressing that also water ice is absorbing at these wavelengths (Figure 1). Nevertheless, we can define a reflectance ratio to estimate the relative depth of the 2.7 μm absorption band (Section 4.1) for use as a general measure of the presence of ice (both H_2_O and CO_2_). This is achieved by taking advantage of orders 169 and 190 (blue and red dashed lines in Figure 1), being the ones with the best spatial coverage in the data set so far available. Order 190 falls between CO_2_ ice absorption bands at 2.28 and 2.35 μm. It also encompasses some gaseous CO absorption lines, but they are narrow enough to not affect the measurement of the continuum.

Toward the thermal range of the LNO spectrum (wavelengths larger than 3.2 μm covered by orders 119–136, see Table 2), the instrumental signal‐to‐noise ratio (SNR) of LNO becomes worse due to a reduced instrumental sensitivity (maximum SNR ∼20, compared to 70 at shorter wavelengths, see below) and, moreover, a limited number of observations are available. This makes the CO_2_ ice absorption bands longward of 3 μm difficult to use at the global scale we are interested in.

As stated above, the combination of orders 193 and 194 covers the 2.28 μm absorption band. However, since a relatively low number of observations are available for these orders, the spatial coverage they provide is somewhat limited. Moreover, the AOTF‐induced spectral mixing (Section 2.2) makes the reconstruction of the full, non‐modulated 2.28 μm band quite challenging.

On the other hand, the absorption band at 2.35 μm (4,255 cm^−1^) is a more promising feature, since it never saturates and is well covered by LNO order 189. This band is already known to be diagnostic of the presence of CO_2_ ice on the surface of Mars (Schmitt et al., 1998) and, as demonstrated through laboratory measurements (Kieffer, 1970) and observations (Larson & Fink, 1972), it is related to a forbidden transition in solid CO_2_ (Calvin & Martin, 1994). It lies in the solar reflected part of the spectrum where the detector sensitivity is higher, thus allowing a higher SNR (70 at max) with respect to longer wavelengths. Moreover, since this band is relatively weak, it is affected to a lesser degree by atmospheric particulate scattering (Section 4.3). However, the moderate SNR of the LNO nadir data set makes its measurement rather noisy and, therefore, we take advantage of a spectral classification method for describing the overall behavior of this band in the data set (see Section 4.2).

**Figure 2:**
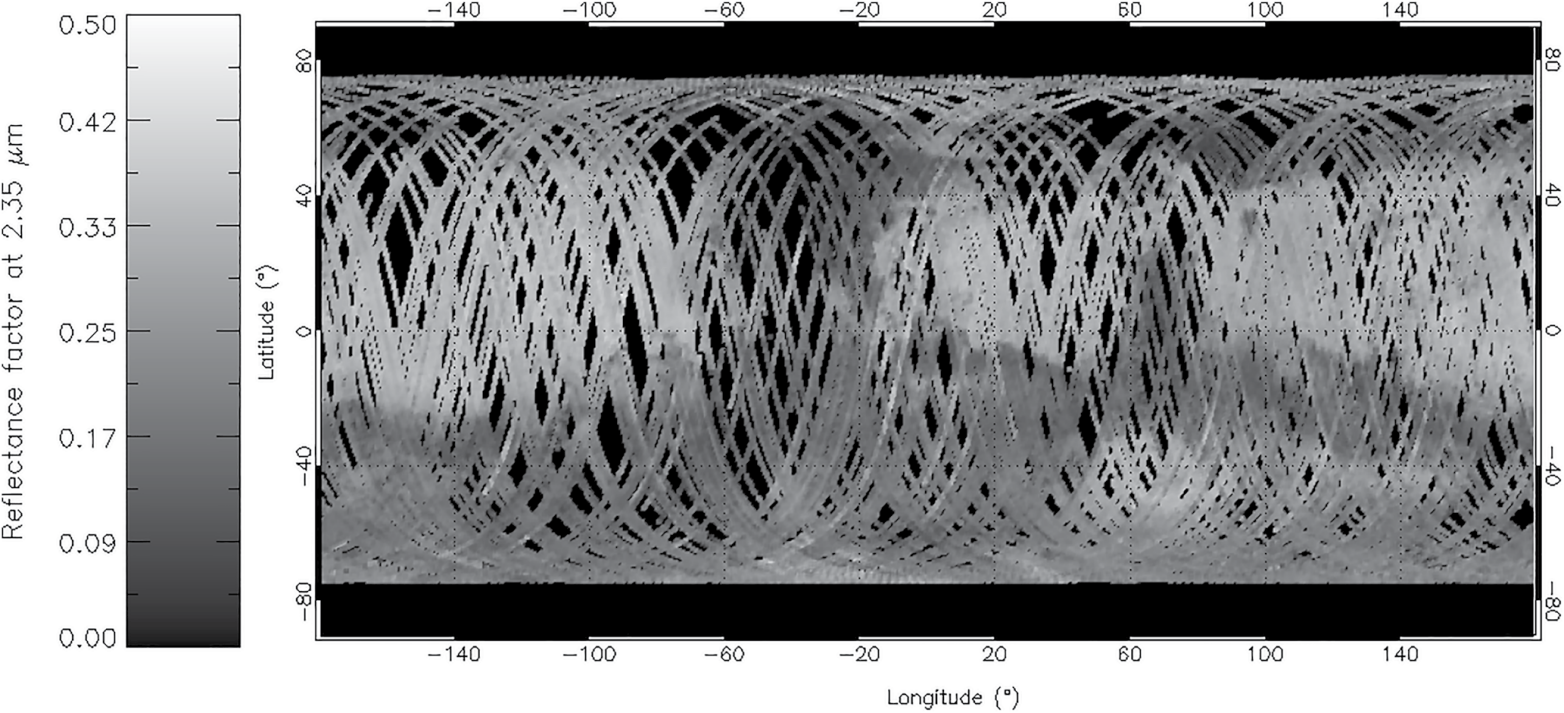
Map of all MY34 (150° < *L*_s_ < 360°) and MY35 (0° < *L*_s_ < 150°) 2.35 μm reflectance factor data of order 189, averaged in bins of longitude and latitude.

For the above reasons, we focus this investigation of the LNO information content about CO_2_ ice on the analysis of the data of orders 169, 189, and 190 (Table 2), to which we apply the Minimum Noise Fraction algorithm (Boardman & Kruse, 1994; Green et al., 1988; Lee et al., 1990) in order to mitigate the noise. Regarding spatial coverage, we point out that the selected NOMAD orders are acquired on consecutive spacecraft orbits, hence they are neither spatially nor temporally coincident. This yields non‐uniform coverage at global scale for each order. In order to work on a common spatial base and reduce possible biases, we reduce all data to the coverage of order 189, which is the most sparse among the others. A more complete spatial coverage can be achieved by accumulating data in a wider range of solar longitudes (Section 4), but with a consequent reduction of the temporal resolution of seasonal trends. A similar issue affects the seasonal coverage too, since the data averaged in bins of latitudes and solar longitudes are acquired at varying longitudes. Taking into account MY34 and MY35 data, the selected orders provide a near‐global coverage of the planetary surface between latitudes ±75° (Figure 2), with southern and northern latitudes mostly covered by MY34 and MY35 data respectively. Local times values vary between early morning and late afternoon, while SZA are moderate at mid‐latitudes (0°–50° on average) and reach night‐time values (SZA > 90°) in the winter pole. However, in order to avoid observations with very low SNR, we select only data with SZA < 80°. The temporal coverage of the data considered here encompasses a full Martian year, from northern summer of MY34 to northern summer of MY35 (solar longitudes 150°–360° in MY34, and 0°–150° in MY35).

It is important to note that the high spectral resolution of LNO data (Table 1) is counterbalanced by a moderate spatial resolution, as the instantaneous footprint on the surface is quite large (0.5 × 17 km, Table 1). This can impact the identification of the ice features, in particular in regions of transition between non‐icy and icy terrains or if observing small ice deposits. In these cases in which the pixel ice filling factor (i.e., the ratio between the instantaneous apparent section of icy terrain and the angular size subtended by the instrument pixel) is less than unity, the ice diagnostic features may be diluted or mixed to different extents with non‐icy spectral features. This fact, in addition to the spectral contamination by AOTF side lobes (Section 2.2), may result in complex deformation in the observed ice spectrum when mixed with other spectral components, making the ice identification less reliable. For the above reasons, we focus our analysis and discussion only in regions where the ice signature can be unequivocally identified (see Section 4.1).

## 3 Spectral Modeling

As stressed in Sections 1 and 2.1, in this work we focus on relative analyses of LNO spectra, whereas more comprehensive radiative transfer studies are postponed to future work (Section 6). This approach makes our results mostly independent from radiative transfer models and calibration issues. Nevertheless, we use simplified radiative transfer models for giving a context to our results and assessing their behavior (Section 5.1). In this section we briefly describe such models, used to perform the simulations of surface ice and dust/ice clouds spectra discussed in Sections 2.3, 4.2, 4.3 and 5.1. For both surface and cloud models, we perform the simulations with SZA = 70° (unless specified otherwise), consistent with NOMAD observing geometry at southern polar latitudes. Moreover, scattering parameters are computed using Mie theory (through the MIEV0 routine, Wiscombe, 1980). The wavelength range we consider is that covered by orders 169, 189 and 190 (Table 2). Given the qualitative purpose of the modeling, we do not apply the Blaze and AOTF functions to the simulations, since they would require a specific characterization depending on the considered order and observation that is not yet fully established (see Section 2.2). For this reason, the models we compare to LNO data in Section 5.1 (Figure 8a) should not be considered as best fits in a retrieval framework, but they still provide valuable information about the physical quantities that, in principle, can be retrieved by means of a more in‐depth radiative transfer analysis of this data set (Section 6).

### 3.1 Surface Ice Albedo Spectra

The albedo spectra of surface ice have been simulated using the SNow Ice and Aerosol Radiation (SNICAR) tool by Flanner et al. (2007), formerly developed for H_2_O snow Earth‐based studies (Singh & Flanner, 2016) and here extended to compute CO_2_ ice albedo using the optical constants from Hansen (2005). The model takes advantage of the multiple scattering, multilayer two‐stream radiative approximation (Toon et al., 1989), with the delta‐hemispheric mean approximation. We adopt a CO_2_ ice mass density of 1,000 kg m^−3^ that is indicative of Mars' south pole (Brown et al., 2010; Titus et al., 2008) and, for models whose only purpose is to showcase the peculiar spectral signatures of surface ice (Section 2.3, Figure 1 and Section 4.2, Figure 5), we adopt the assumption that the thickness of the ice slabs is equal to the grain size (Langevin et al., 2007). However, such an assumption is not realistic in general since in the considered spectral range we are only sensitive to the upper layers of surface ice deposits. For this reason, in order to compare the models to LNO observations in a semi‐qualitative way (Section 5.1) we vary the slabs' optical depth by changing both their thickness and size.

### 3.2 Airborne Dust and CO_2_ Ice Clouds Reflectance Factor Spectra

The reflectance factor of Martian dust layers and CO_2_ ice clouds is obtained through the MITRA radiative transfer tool (Adriani et al., 2015; D’Aversa et al., 2022; Oliva et al., 2016, 2018; Sindoni et al., 2017), already applied to the atmospheres of Saturn, Jupiter and Mars. Dust layers are simulated using the optical constants from Wolff et al. (2009, 2010) while for CO_2_ ice clouds we adopt the refractive index defined by Hansen (2005), as in the case of surface ice modeling. Dust layers and ice clouds are described adopting lognormal particle size distributions with 0.5 effective variances (parameters taken from Mars Climate Database, MCD, Millour et al., 2019). Regarding dust opacity, at the wavelengths considered in this work we are more sensitive to abundance than grain size variations and, hence, the latter parameter is assumed constant (1 μm effective radius) in all the simulations. Given the considered spectral range, gaseous absorption has no impact for our purpose and, for this reason, it is not considered in the computations. In Section 5.1, where we compare our models to LNO data, we adopt their specific illumination and observing conditions, and use the method from McGuire et al. (2009) on OMEGA data to estimate the spectral surface albedo required to perform the simulations.

## 4 Data Analysis

Two different approaches were used to identify and map the CO_2_ ice on the surface: (a) the analysis of the short‐wavelength shoulder of the 2.7 μm band, through orders 169 and 190 investigation (Section 4.1) and (b) the application of a spectral matching method to the shape of the 2.35 μm band (Section 4.2).

### 4.1 Ice Detection Through the 2.7 μm Band

As already explained (Section 2.3) the 2.7 μm band is usually saturated at the expected conditions for surface CO_2_ ice on Mars. Moreover, given the band strength, it is also quite sensitive to atmospheric particulate scattering (Vincendon et al., 2008; see Section 4.3). For the above reasons, albeit this band is not reliable to quantify the amount of ice on the surface without a proper radiative transfer analysis, or for its detection in no‐ice to ice transition regions (Section 5), it can be exploited to identify spatially homogeneous and relatively abundant deposits. Therefore, the simplest indications of ice detection can rely on coupling a high absolute reflectivity level of the spectrum at continuum wavelengths with a stronger absorption inside the 2.7 μm band. We define the Ice Index as the ratio between the reflectances measured in orders 190 and 169 (Section 2.3):

(2) $$Ice\;Index=\frac{R_{190}}{R_{169}}$$

**Figure 3:**
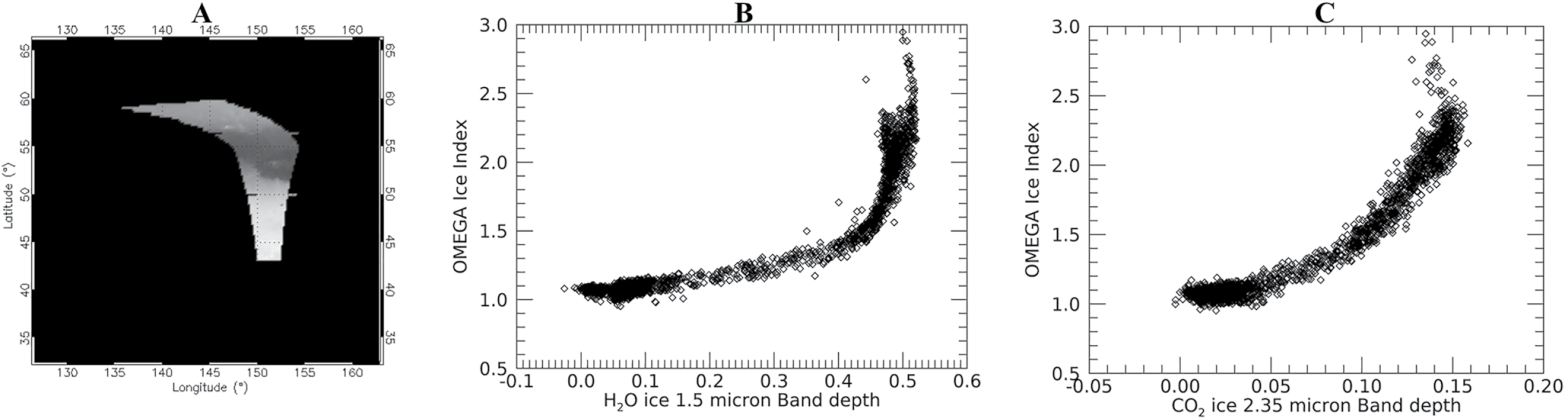
Cylindrical projection of a cut of OMEGA orbit 0228_3, covering mid to polar Northern latitudes during MY27, displayed at 2.33 μm (panel a). Panels (b and c) show the trend of the OMEGA Ice Index computed on OMEGA orbit 0228_3 (panel a) with the 1.5 μm H_2_O ice band depth (b) and the 2.35 μm CO_2_ ice band depth (c).

This ratio is of course applicable also to other datasets, for example, for OMEGA, that we use here as a benchmark to test its effectiveness in detecting ice. As we can clearly see in Figure 3, the Ice Index calculated for OMEGA spectra (for OMEGA, Equation 2 reduces to the simple ratio of reflectance factors at 2.62 and 2.33 μm) correlates with CO_2_ ice abundance, measured through the depth of the 2.35 μm band (panel c in the figure). This test also confirms the Ice Index to be sensitive to H_2_O ice, measured through the depth of the 1.5 μm band (panel b in the same figure). As expected, the Ice Index has a value of about 1 on non‐icy terrains while it becomes larger for stronger band depths. Since the Ice Index is evaluated on the 2.7 μm band shoulder, different growth regimes versus the band depth are visible, depending on the width and shape of the considered H_2_O and CO_2_ ice absorption bands. Its slow growth rate with smaller band depths makes its uncertainty larger in smaller or more dusty ice deposits. Nevertheless, it is evident how, aside from its uncertainty in transition regions, the Ice Index can be reliably used to identify deposits where the ice abundance (and hence the band depth) is large. In the OMEGA case, the possibility to directly measure the band depths, although at moderate spectral resolution, enables each pixel to be flagged as icy or not and consequently to potentially derive threshold values for ice detection based on the actual uncertainty associated with the OMEGA data. However, this approach cannot be extrapolated to the NOMAD data set, since the differences in data acquisition and processing affect the threshold values.

With NOMAD data we are interested in longitudinally averaging the Ice Index, in order to perform a seasonal study of the ice coverage. To achieve this, we group the reflectance factors taken at the central wavelengths of the selected orders (*R*_169_ and *R*_190_, taken at 2.62 and 2.33 μm respectively) in bins of latitude and Ls, by averaging the data at all available longitudes. As a result, we obtain a latitudinal‐seasonal map for each order. As mentioned in Section 2.3, a different longitudinal coverage pertains to orders 169 and 190, making their direct ratio biased by spatial albedo inhomogeneities. In order to mitigate this issue we resort to MGS/TES data on Mars bolometric albedo (Christensen et al., 2001). By keeping track of the longitudes falling in each bin of the two orders' seasonal maps, we average TES data the same way as NOMAD ones, obtaining a corresponding map for each order. We can then use these maps as weighting factors to correct for the different spatial coverage of the two orders. We define the zonally averaged index <Ice Index> that is sensitive to the presence of ices, namely:

(3) $$<Ice\;Index>\;=\frac{R_{<190>}}{R_{<169>}}∙\frac{{TES}_{<169>}}{{TES}_{<190>}}$$

where *R*_<*X*>_ and TES_<*X*>_ are the zonally averaged NOMAD and TES seasonal maps respectively, and _<*X*>_ denotes the average along the longitudes inside the same latitude/*L*_s_ bin of order _*X*_. The ratio of the reflectance in the two orders, weighted by TES albedo data, correlates with the amount of ice in the footprint.

**Figure 4:**
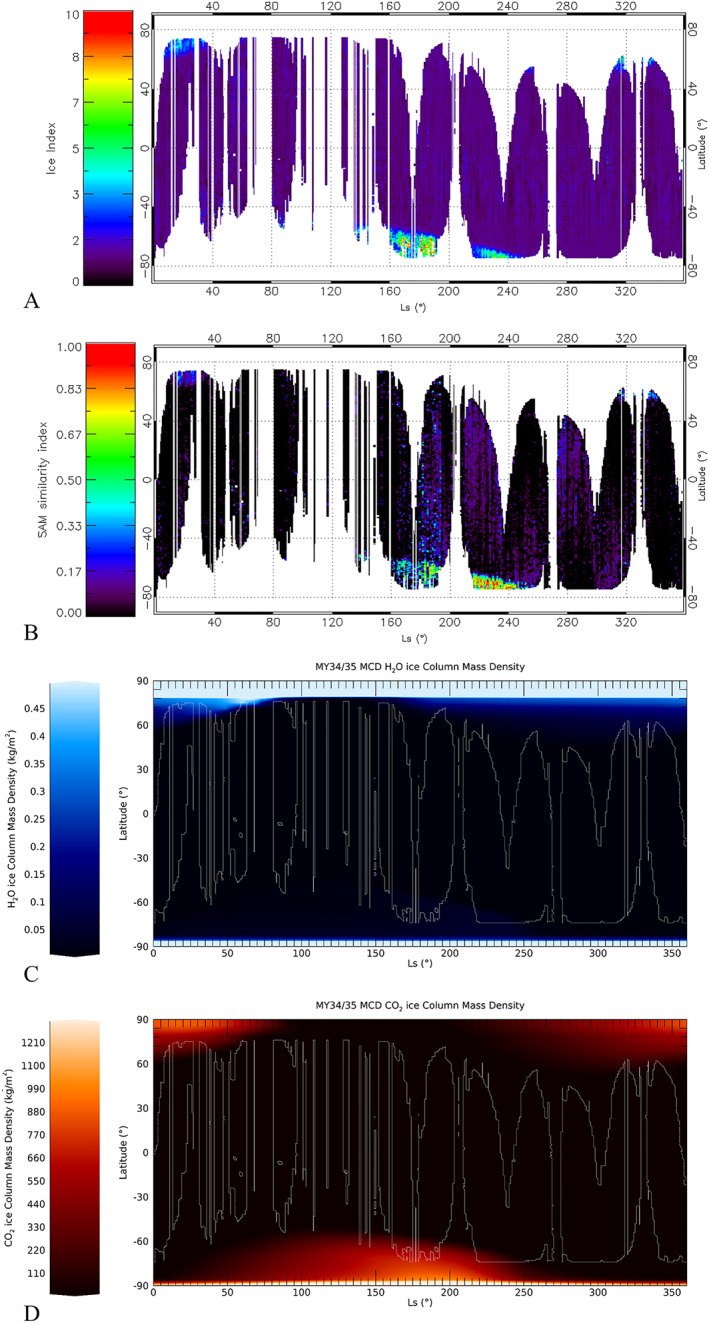
In all maps, Ls between 0° and 150° refer to MY35, while Ls between 150° and 360° are related to MY34. The image shows latitudinal‐seasonal maps of Ice Index (a), Spectral Angle Mapper (SAM) similarity index *χ* = 1−*α*/*α*_mid_ (panel b, α_mid_ is the average SAM angle α estimated at mid‐latitudes, see Section 4.2.1) and (c) MCD predictions of H_2_O and (d) CO_2_ ices columnar mass density in kg/m^2^. The boundaries of the Nadir and Occultation for MArs Discovery data set as shown in panels (a and b) are superimposed on panels (c and d) for a qualitative comparison.

It is evident how the approach in the computation of the Ice Index with OMEGA and NOMAD datasets is quite different. Significant differences between the two datasets come from the relatively high LNO noise level, its spectral resolution, the related penetration depth in the ice layers, and the TES data uncertainty. Regarding this last issue, we remind the reader here that TES albedo is bolometric, and therefore carries information related to a wider spectral range with respect to the single wavelengths we are considering with NOMAD data. Moreover, despite the TES map being filtered to minimize the effect of atmospheric dust and clouds, it still contains surface ice (Christensen et al., 2001), increasing the uncertainty in transitional regions. Another difference between OMEGA and NOMAD datasets is that OMEGA spectral resolution is lower (resolving powers of 10^2^ and 10^4^ for OMEGA and NOMAD respectively). This implies that the OMEGA steep short wavelength shoulder of the 2.7 μm band is probably probing different depths of penetration in the ice with respect to NOMAD, due to the wider wavelength range convolved in a single spectral point. All these sources of uncertainty are particularly significant in ice/no‐ice transition regions, and, overall, prevent the definition of an absolute threshold value for ice detection. For this reason, we focus on the global distribution of the Ice Index and its qualitative correlation with regions of interest on the planet. As in the case of OMEGA, in the NOMAD data set Ice index values as low as 1 are found in regions where no large concentrations of ices are expected, for example, at the equator and mid‐latitudes. On the other hand, an Ice Index larger than 2 is found on the polar caps where both H_2_O and CO_2_ ices are known to be abundant (Figure 4a). Values between 1 (ice free terrains) and 2 (polar caps) possibly still indicate the presence of ices in transition regions with smaller ice filling factors, making it difficult to obtain an unambiguous detection. The same effect could be produced by dust areal mixing on the ice slabs, as observed by Langevin et al. (2006) on the “cryptic” region (latitudes < −70°) of the South polar cap. However, due to the averaging procedure described above and to LNO spatial resolution (Table 1), the resulting seasonal data set we analyze here is poorly sensitive to such region. Instead, in regions where the Ice Index is larger than 2 the pixels are more likely to contain mostly ice and, hence, its information is more consistent. It is worth stressing again that this value should not be considered as a threshold between non‐icy and icy terrains, but rather an indication for abundant deposits in which the ice spectral signature is stronger. The terrains identified with Ice Index > 2 mostly fall within Mars polar caps but a few regions are also located at mid‐latitudes (Section 5.1).

### 4.2 CO_2_ Ice Detection Through the 2.35 μm Band

**Figure 5:**
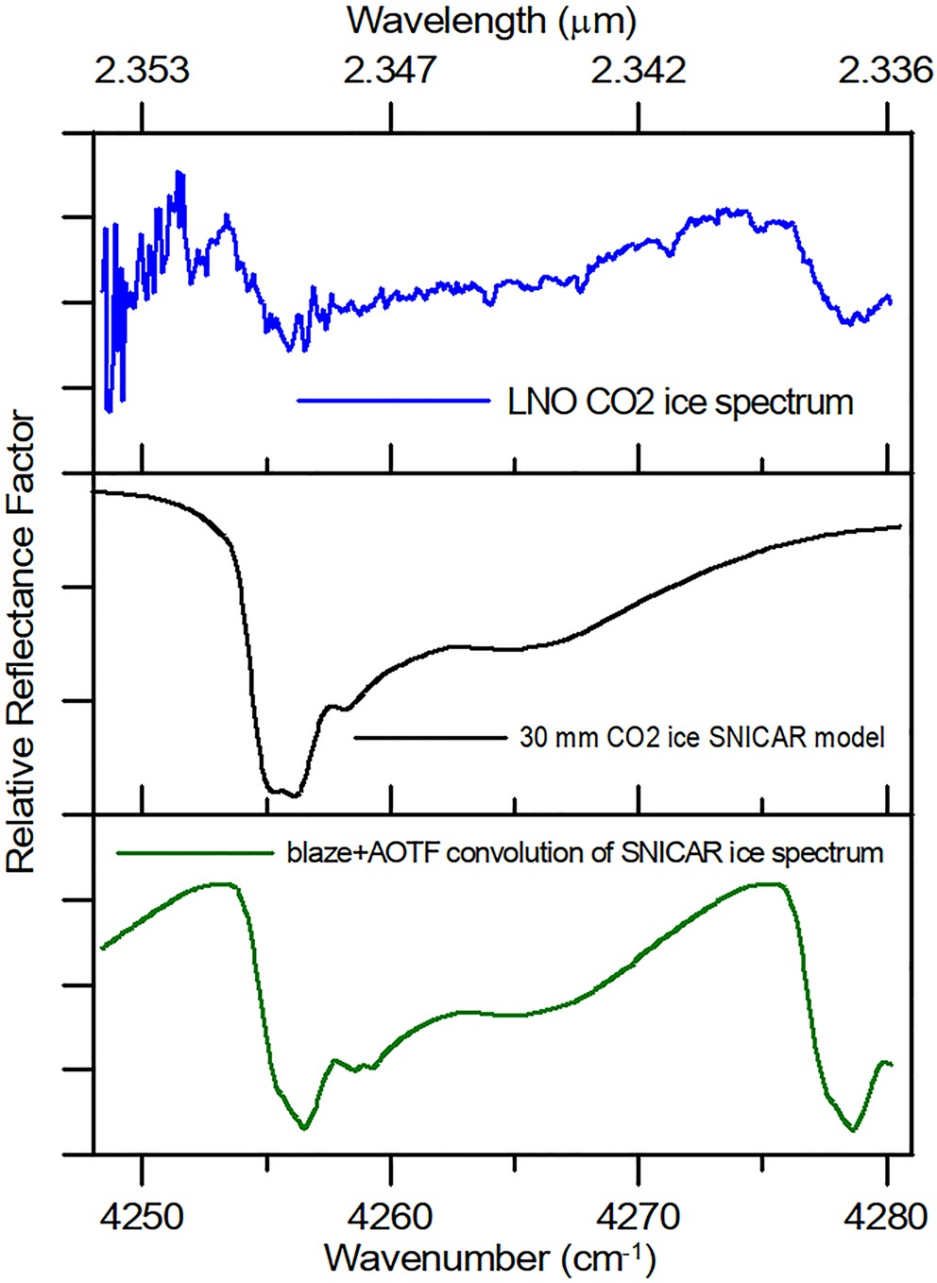
Blue: Nadir and Occultation for MArs Discovery (NOMAD) reflectance spectrum of the South polar region. The CO_2_ ice absorption is visible at 2.350 μm (4,255 cm^−1^). Black: model spectrum of a 30 mm thick CO_2_ ice slab obtained with the SNICAR tool (Section 3.1). Green: the black spectrum convolved with the NOMAD instrumental functions (the Blaze and AOTF response).

As described in the previous section, the Ice Index is sensitive to both CO_2_ and H_2_O ices. An independent method is therefore desirable in order to distinguish them. The 2.35 μm band of CO_2_ ice is covered by the LNO order 189. The icy spectra in this order show a stronger absorption band centered at about 2.350 μm (4,255 cm^−1^) embedded in a wider absorption between 2.339 μm (4,275 cm^−1^) and 2.351 μm (4,253 cm^−1^; an example is given in Figure 5). The spectral shape measured by LNO is affected by the AOTF side‐lobes contamination (Section 2.2), which results in a ghost absorption band appearing at 2.337 μm (4,278 cm^−1^). Since the width of the 2.35 μm absorption band is of the same order of the AOTF bandwidth, the measurement of the full band depth is not possible, as the continuum level of the reflectance is hardly determined. Moreover, the SNR at the edges of the order, at wavelengths shorter than 2.337 μm and longer than 2.351 μm drops significantly. The definition of a pseudo‐continuum between 2.339 μm (4,275 cm^−1^) and 2.351 μm (4,253 cm^−1^) will allow the measurement of a partial band depth which cannot quantify the amount of CO_2_ ice (since its continuum is still affected by CO_2_ ice absorption) but it is qualitatively correlated to its abundance. However, the uncertainties associated with such parameters are still large, since they always deal with the LNO signal measured in few spectral channels.

#### 4.2.1 Spectral Angle Mapper Analysis

In order to obtain a more robust parameter, able to statistically map the CO_2_ ice detection with LNO, we use the Spectral Angle Mapper (SAM) method (Boardman, 1992; Kruse et al., 1993). The SAM algorithm determines the spectral similarity between a sample spectrum and reference ones by treating them as vectors in a space with dimensionality equal to the number of considered bands and by calculating the correlation, expressed by the angle α (in radians) between them. Hence, the smaller the angle, the better the agreement with the considered reference spectrum. This technique, when used on calibrated reflectance data, is relatively insensitive to illumination and albedo effects (Kruse et al., 1993), and is also effective in mitigating the propagation of uncertainties since it involves the whole central part of the spectral range.

We apply the SAM algorithm to the LNO spectra of order 189 between 2.337 μm (4,278 cm^−1^) and 2.351 μm (4,253 cm^−1^). As a reference endmember, we adopt a bulk southern polar cap spectrum in which the 2.35 μm CO_2_ ice absorption band is clearly recognizable and whose spectral shape is in agreement with that expected from radiative transfer models (Figure 5). Before the application of the algorithm, we apply the same spatial averaging procedure described for the Ice Index (Section 4.1), in order to obtain a spectral array in bins of latitudes and solar longitudes. Then, we then define a SAM index of spectral similarity as *χ* = 1−*α*/*α*_mid_ (where *α*_mid_ is the average angle estimated at mid‐latitudes where surface ice is unlikely to be present). The resulting map of the SAM similarity index *χ* is shown in Figure 4b. We tested different endmembers, finding no significant difference in the SAM results as long as the endmembers are taken in the bulk of the polar caps, where the mixing with non‐icy terrains is likely negligible. On the other hand, endmembers taken at the borders of the cap result in noisy SAM maps, where the polar caps are not even detectable.

#### 4.2.2 Ice Index and SAM Seasonal Maps Qualitative Comparison

It is evident how the SAM map in Figure 4b is noisier with respect to the Ice Index one in Figure 4a, due to the fact that order 189 has a more limited spatial coverage with respect to orders 169 and 190, used for the Ice Index computation, hence yielding a poorer SNR in the zonal averages. This is particularly evident in the pre‐storm period (180° < *L*_s_ < 200°) at mid‐latitudes, where the relative errors of the Ice Index and SAM similarity index are maximum and reach 30% and 50% respectively. Nevertheless, we note that the polar caps regions correlate with χ values larger than 0.2 (Figure 4b). As in the case of the Ice Index (Section 4.1), we stress that this value should not be considered as a threshold for the CO_2_ ice detection, but rather an indication of deposits where the ice abundance is large and the 2.35 μm band signature strong. Such *χ* values also identify several non‐polar latitude regions that are discussed in Section 5.1.

We can now qualitatively compare the seasonal Ice Index (Figure 4a) and SAM *χ* index (Figure 4b) maps with the simulations of seasonal H_2_O and CO_2_ ices abundance, expressed as columnar mass density in kg/m^2^ (Figures 4c and 4d respectively) obtained from the MCD (version 6, Millour et al., 2019). In these simulations, airborne dust climatology is treated with the method described in Montabone et al. (2020). Both the Ice Index and the SAM maps are effective in reproducing the condensation and sublimation patterns taking place in the Martian polar caps. In particular, during the Northern spring (*L*_s_ ∼ 0°) the sublimation of the northern polar cap is observed in both maps, in agreement with MCD simulations. Although fewer observations are available for southern winter (*L*_s_ ∼ 90°), we observe some clear detections of the southern polar cap in the Ice Index map. These are in agreement with the presence of both H_2_O and CO_2_ ices and, since no coincident detections are available in SAM map, we cannot determine which ice is being probed by the Ice Index. During the southern late winter/spring we observe the sublimation of the southern polar cap both in the Ice Index and SAM maps (140° < *L*_s_ < 260°). This solar longitude range is also affected by the 2018 global dust storm (*L*_s_ ∼ 180°–250°, Smith & Guzewich, 2019; Viúdez‐Moreiras et al., 2019) allowing investigations of the impact of dust on the ice detection (Section 5). The SAM map is noisier than the Ice Index map before the peak of the storm (160° < *L*_s_ < 200°), due to the scarce data coverage and poor SNR of order 189 data near the poles in the illumination conditions typical of late winter. During the decay phase of the storm (*L*_s_ ∼ 200°–250°) both maps correctly reproduce the sublimation pattern of the ice caps predicted by the MCD.

Finally, a few detections appear during northern autumn and winter (*L*_s_ > 255°), indicating the condensation and the peak winter phase of the northern polar cap. In this period, ice is identified in both the SAM and Ice Index maps only for *L*_s_ > 280°. On the other hand, at *L*_s_ ∼ 255° only Ice Index detections are found, suggesting that the parameter is probably probing H_2_O ice at those solar longitudes. The dichotomy between the SAM and the Ice Index map makes it possible to distinguish the presence of H_2_O and CO_2_ ices and is discussed further in Section 5.

### 4.3 Airborne Dust Effect

**Figure 6:**
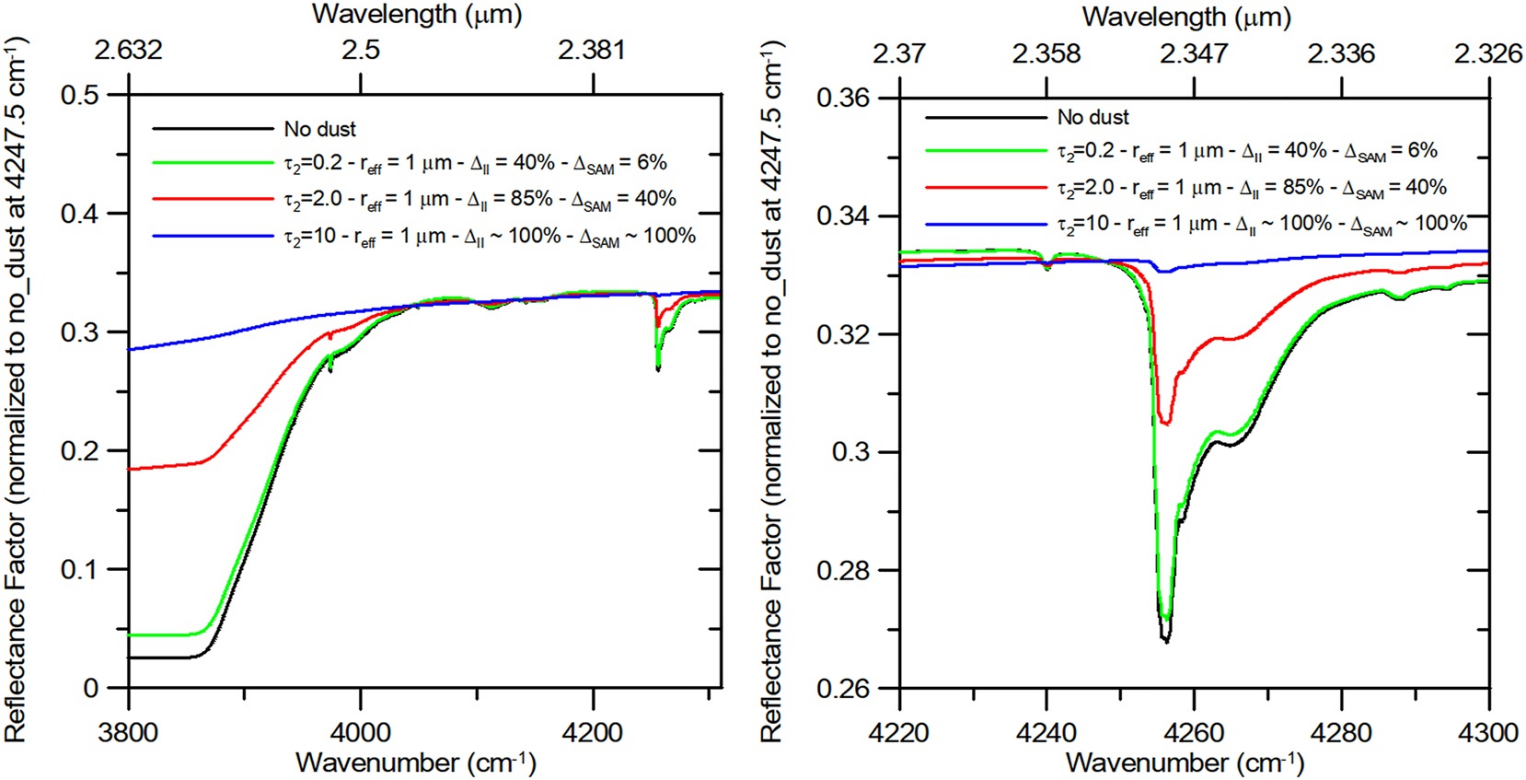
Radiative transfer simulations performed with the MITRA tool (Section 3.2) adopting different optical depths (*τ*_2_ is the optical depth at 2 μm). Δ_II_ and Δ_SAM_ indicate the percent decrease of the Ice Index and Spectral Angle Mapper similarity index with respect to the *no dust* simulation (black lines). All simulations have been normalized to the *no dust* one at 4247.5 cm^−1^. The CO_2_ ice albedo underneath the dust is computed with the SNICAR tool (Section 3.1), assuming a 30 mm slab. Left panel shows a wide spectral range including both the 2.35 μm and the broader 2.60 μm CO_2_ absorptions, while the right panel shows the zoom on the 2.35 μm band.

The CO_2_ ice detection approach described in Sections 4.1 and 4.2 is based on the assumption that suspended dust has a negligible impact on the detection of CO_2_ ice spectral features. We verify this assumption by performing radiative transfer simulations with different dust optical depths in the wavelength range covered by orders 169, 189, and 190 (Table 2). We compute the CO_2_ ice surface spectral albedo using the SNICAR model (Section 3.1) and the MITRA radiative transfer tool to simulate the Martian reflectance factor with different dust loadings (Section 3.2). The results are shown in Figure 6. The presence of dust decreases the relative depth of the CO_2_ ice absorptions at 2.60 and 2.35 μm, while changes in spectral shape happen only for very high abundances, as it is evident in the blue simulations in which *τ*_2_ = 10 (where *τ*_2_ is the dust optical depth at 2 μm). As a consequence, dust lowers the Ice Index value (Section 4.1) affecting its effectiveness in the identification of ice. Ordinary dust abundances (*τ*_2_ < 1) can impact the detection of small/thin ice deposits through the Ice Index, but conditions of high dust optical depth can even mask extended icy surfaces. Indeed, with respect to the clear sky case, we estimate a decrease of the Ice Index down to 85% with *τ*_2_ = 2. This analysis confirms that, even if it is beyond the scope of this paper (Section 5), the CO_2_ ice 2.7 μm absorption band can be used to estimate the atmospheric dust content, as already done with OMEGA data by Vincendon et al. (2008). It is worth stressing that the vertical distribution of dust is not constrained in these simulations, so the same effect holds either for dust suspended in the whole atmospheric column, concentrated in an atmospheric layer or directly deposited over the surface ice.

On the other hand, dust has a lesser impact on the CO_2_ ice identification through the SAM similarity index χ (Section 4.2). This is expected from a theoretical point of view because the 2.35 μm band is relatively weak and its depth is less affected by scattering. Moreover, the SAM algorithm is more sensitive to the band shape than to its depth. Therefore, the ice identification is still possible as long as the 2.35 μm absorption band is above the noise level. The relatively low SNR of order 189 is the most limiting factor in the effectiveness of the SAM algorithm application. The percent decreases of both the Ice Index and SAM similarity index due to the dust presence with respect to the clear sky case (Δ_II_ and Δ_SAM_ respectively) are given in Figure 6.

## 5 Results and Discussion

As described in Section 4, if we focus on regions where ice is abundant, that is, the polar cap regions, the Ice Index and SAM seasonal maps of surface ice coverage are in good agreement with general climate models (Figure 4). It should be noted that polar caps observations are unavoidably acquired at high solar zenith angles (between 50° and 80°). Such geometry yields unfavorable illumination conditions in which both the incident and reflected radiation are reduced in intensity and topographic shadowing is amplified, implying increased average mixing of illuminated and unilluminated areas and reduced SNR. This issue affects both the Ice Index and the SAM maps, possibly enlarging those regions that we identify as transition between icy and non‐icy terrains.

**Figure 7:**
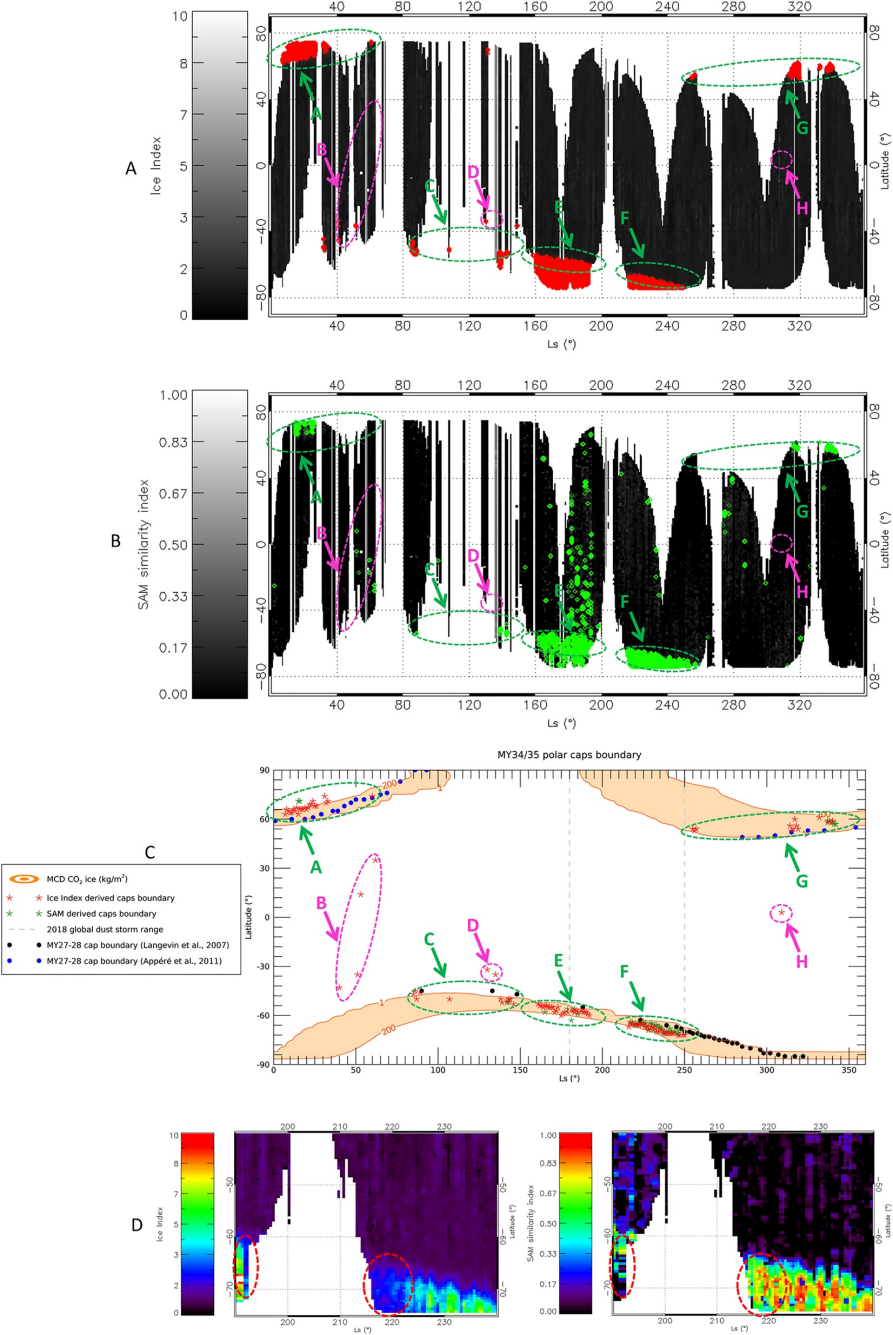
In all panels, Ls between 0° and 150° refer to MY35, while Ls between 150° and 360° are related to MY34. Panels (a and b) Ice Index (a) and Spectral Angle Mapper (SAM) *χ* (b) maps where red and green points indicate regions with Ice Index >2 and SAM index *χ* >0.2 respectively (see Section 4.1 and Section 4.2). Colored labeled arrows indicate structures of interest identified in panel (c), on the polar caps (green) and at mid‐latitudes (magenta, Section 5.1). Panel (c) red and green stars indicate the polar caps boundaries estimated through Ice Index and SAM similarity index *χ* respectively (Section 5). These are compared to the MCD simulated abundances of CO_2_ ice corresponding to the observed cap boundaries positions (orange regions, representing surface column mass densities in kg/m^2^) and to OMEGA observations (black and blue dots for the South and North poles respectively) from MY27‐28 (Appéré et al., 2011; Langevin et al., 2007). The dashed gray lines indicate the 2018 global dust storm *L*_s_ range. Panel (d) comparison of the South polar cap in the Ice Index and SAM χ index maps within the storm *L*_s_ range. The red dashed circles indicate regions in which the dust storm strongly impacts the Ice Index value.

The noise level in the SAM *χ* index map is higher with respect to the Ice Index map (Section 4.2.2, Figure 4). As a result, some structures identified in the latter are not recognizable in the former. This is evident in Figures 7a and 7b, where the red and green points indicate pixels with Ice Index >2 (Section 4.1) and SAM *χ* index >0.2 (Section 4.2) respectively, highlighting regions in which the two parameters mostly correlate with the polar caps. In particular, the SAM map misses some points at the North pole in regions A and G. This is in agreement with the fact that CO_2_ ice requires colder temperatures to condense and, hence, suggests that it is probably located at latitudes higher than those covered in the analyzed data set. The mid latitude detections in the SAM map are discussed in Section 5.1.

From the Ice Index and SAM maps we can estimate the extension of the polar caps by studying the latitudinal trend of the two parameters for every bin of *L*_s_. We define the edge of a cap at a given Ls as the latitude of the most equatorward group of at least three adjacent pixels in which the parameters exceed by three sigma their average value on mid‐latitudes non‐icy terrains. Such a condition has been chosen to ensure ruling out the aforementioned ice transitional regions, where filling factor issues can make ice parameters values unreliable (Sections 2.3 and 4.1). The results we obtain are compared in Figure 7c to the MCD CO_2_ ice abundance simulations and to OMEGA data estimated boundaries for MY27‐28. In the image, red and green stars indicate the Ice Index and the SAM estimated boundaries respectively, while the dots represent the minimum latitudes in the polar caps at which OMEGA 1.43 μm CO_2_ ice absorption band is non‐zero, extrapolated from Figures 12–19 from Langevin et al. (2007) and from Figures 4–9 from Appéré et al. (2011). The colored regions of interest labeled with letters identify the polar caps (green regions) and mid latitude ice detections (magenta regions). For comparison, these are also shown in Figures 7a and 7b. It must be noted that there is not always coincidence between these regions and the colored points in Figures 7a and 7b (see e.g., region H), being their identification based on conceptually different conditions.

Since our boundary estimates are not sensitive to transition regions, the comparison we make with MCD is merely qualitative and no attempt to perform a quantitative retrieval of the ice abundance is being made. The MCD CO_2_ ice column density isolines shown in Figure 7c are those coincident with our cap boundaries estimates and highlight some gradients in abundance across the edges that are worth discussing. OMEGA boundaries are consistent with our estimates and with the lower density MCD simulations, confirming the seasonal patterns of the polar caps in different MY. As in the case of the Ice Index and SAM points in Figures 7a and 7b, there is not always correspondence between the red and the green stars clusters in Figure 7c. This is particularly evident in regions C and G, in which the SAM boundaries only appear in coincidence with the highest *L*_s_ Ice Index clusters. As explained above, this behavior is related to the fact that, in these regions, CO_2_ ice is probably condensing at lower temperatures pertaining to latitudes higher than those covered in our analysis. Indeed, in region A the SAM boundary appears farther north with respect to the Ice Index boundary. The MCD predicts average surface temperatures of about 160 K for the red stars cluster in region A, above the CO_2_ frost temperature, suggesting that the Ice Index points are actually probing the H_2_O ice polar cap there. On the other hand, temperatures of the order of ∼150 K are predicted for the green stars cluster, within Mars CO_2_ frost temperature range (Piqueux et al., 2016) and in agreement with the presence of the CO_2_ ice points. In all regions where green clusters overlap on the red ones (regions E, F, and at *L*_s_ ∼ 140° in region C and *L*_s_ ∼ 340° in region G), the Ice Index is probing both H_2_O and CO_2_ ices. On the other hand, in the lower *L*_s_ clusters in regions C (85° < *L*_s_ < 105°) and G (255° < *L*_s_ < 320°) only Ice Index derived boundaries appear, suggesting that the parameter is only probing H_2_O ice there. The estimated cap boundaries show a drift to larger MCD CO_2_ ice column densities from the peak phase to the sublimation phase of both the North (regions G to A) and the South (regions C, E and F) polar caps. Such a trend may be related to non‐uniform sublimation processes (enlarging ice‐free areas embedded in icy terrains at several spatial scales), possibly yielding an increase of the areal mixing of terrains within the NOMAD footprint, leading to the concept of inner/outer crocus line (Schmidt et al., 2009). This is consistent with the spatially inhomogeneous sublimation known to take place in Mars seasonal caps (Cull et al., 2010; Hansen et al., 2013; Piqueux et al., 2003; Schmidt et al., 2009) and is also observed in OMEGA data during the sublimation phase of the North polar cap (region A, Figure 7c).

As a consequence, our boundary detection condition is close to the inner crocus line, that is, where the ice abundance is still large and its spatial distribution more homogenous (this may also explain why some of the green clusters are missing in the considered data set, see above).

On the other hand, OMEGA boundaries (black and blue dots in Figure 7c) are consistently located at latitudes lower than those pertaining to the Ice Index and SAM estimated ones and, hence, closer to the outer crocus line.

The data we are considering also partially cover the evolution of the 2018 global dust storm (vertical dashed gray lines in Figure 7c), from its maturation (*L*_s_ ∼ 180°–190°) to its decay phase (*L*_s_ ∼ 203°–250°, Smith & Guzewich, 2019; Viúdez‐Moreiras et al., 2019). Figure 7d compares the Ice Index (left) and SAM (right) maps from Figures 4a and 4b zooming them within the storm Ls range, and shows how the two parameters behave across the storm at the southern pole. While there is a gap of observations during the peak phase, the impact of the storm on the Ice Index map around *L*_s_ ∼ 220° can be seen (dashed red circle). In this region, its value decreases significantly, while the same does not happen in the SAM similarity index map. A similar behavior is observed at *L*_s_ ∼ 190°, during the storm peak phase, where the Ice Index experiences a sudden drop just before the observation gap. This is as expected, as demonstrated in Section 4.3, since the strength of the 2.7 μm band makes it strongly sensitive to the presence of dust, unlike the 2.35 μm absorption band which is weaker (Figure 6). Despite this drop in the Ice Index, the parameter successfully identifies the ice in the polar cap at those solar longitudes (Figures 7a and 7c). Unfortunately, the gap in observations prevents us from verifying if the Ice Index fails in spotting the ice when a very high dust optical depth is reached during the storm peak phase. Under the same assumptions made in Section 4.3 for the modeling of dust, a decrease of the Ice Index as that observed in the highlighted regions in Figure 7d (about 50% with respect to the surrounding regions in the southern polar cap) would indicate a dust optical depth *τ*_2_ ∼ 0.5. Such an approach indicates that an estimation of the dust abundance through the Ice Index is in principle possible (see Section 4.3). However, in order to be quantitatively feasible, this would require a dedicated radiative transfer analysis of both surface ice and airborne dust distribution and microphysical properties that is beyond the scope of this paper.

**Figure 8:**
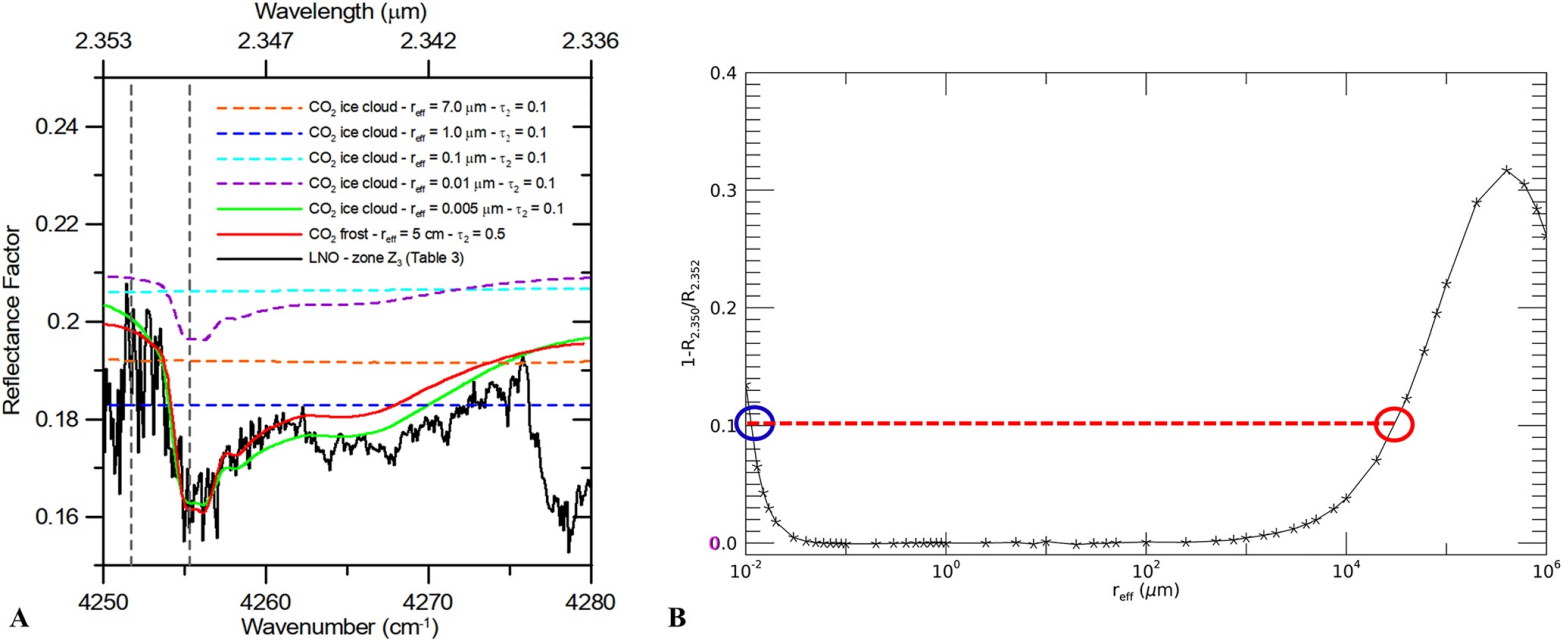
(a) Example of CO_2_ ice spectrum from region Z3 in Table 3, showing the 2.35 μm CO_2_ ice band (solid black line) compared with CO_2_ ice clouds simulations (Section 3.2) with different grain sizes (orange, blue, cyan, purple dashed lines, and solid green line and for *r*_eff_ = 7, 1, 0.1, 0.01, and 0.005 μm respectively, *τ*_2_ is the optical depth at 2 μm). A 5 cm surface CO_2_ ice simulation is also shown (solid red line). Vertical dashed gray lines indicate the wavelengths adopted to quantify the left shoulder slope of the 2.35 μm band as 1−*R*_2.350_/*R*_2.352_ (*R* is the reflectance factor and the subscripts indicate the wavelength in μm). (b) Trend of 1−*R*_2.350_/*R*_2.352_ with the CO_2_ ice effective radius, for a fixed optical depth *τ*_2_ = 0.4. The red dashed line indicates the order of magnitude of the slope values measured in the observed spectra (about 10%), in agreement with the synthetic ones in the 1–10 nm and 1–10 cm size regimes (blue and red circles respectively).

Some isolated ice detections from the Ice Index map appear in non‐polar regions and are identified in magenta in Figure 7c. These detections cover latitudes between 40°S and 40°N in regions B and D and even approach the equator in region H. A faint 2.35 μm absorption band is recognizable in all these observations (Figure 8), confirming the presence of CO_2_ ice. The discussion of these cases is presented in the following Section 5.1.

### 5.1 Non‐Polar Ice Detections

As shown in Figures 7a and 7b, some high values of Ice index and SAM *χ* index are found not only at the polar caps but also in bins at lower latitudes. Most of these detections (some of which are false positives due to poor SNR in the longitudinal averages, in particular in the SAM map in the range 180° < *L*_s_ < 200°, see Section 4.2.2) are filtered out by the condition we adopted for the identification of the cap boundaries, described in Section 5. However, some of those related to the Ice Index still appear in Figure 7c (magenta regions). The presence of ice in longitudinal averages at these latitudes cannot easily be explained, but the analysis of the distribution of the individual spectra averaged within those bins reveals that these detections are driven by single pixel occurrences of icy spectra. These individual spectra of LNO order 189 all show the 2.35 μm absorption band confirming the identification of CO_2_ ice in these regions (zones B–H in Table 3). Given this finding, we similarly scan the single pixel observations falling in the bins highlighted in green in Figure 7b and find other observations at mid‐low latitudes in which the CO_2_ ice signature can be clearly identified (Figure 8a, zones Z in Table 3). Of course, with such an approach we could actually be missing other observations in which the ice signature is present, since the method described in this paper is mostly effective for spatially extended and abundant ice deposits where the ice signature is strong (Sections 2.3, 4.1 and 4.2). Since the ice filling factor in these pixels is not known it is not possible to estimate the spatial extension of these ice deposits within the LNO footprint (Table 1).

**Table 3:** Dates, Longitudes (Lon), Latitudes (Lat), Solar Longitudes (*L*_s_), Local Times (Lt), and MCD Estimated Surface Temperatures (*T*) of the Non‐Polar CO_2_ Ice Detections Identified From Regions B, D, and H in Figure 7c and From the SAM Map (Figure 7b) Green Bins Analysis (Zones Z, Section 5.1)

Non‐polar CO_2_ ice zone	Observation date (yy/mm/dd)	Lon (°)	Lat (°)	*L*_s_ (°)	Lt (h)	*T* (K)
B_1_	2019/06/19	−42.3	−46.2	41.5	10.5	190
B_2_	2019/07/11	−157.1	−37.8	51.3	15.8	206
B_3_	2019/07/14	−164.8	15.9	52.7	16.3	243
B_4_	2019/08/02	164.8	33.8	60.8	12.8	277
D_1_	2020/01/05	134.3	−27.6	131.1	15.2	239
D_2_	2020/01/17	12.09	−31.6	136.7	12.8	249
H	2018/12/21	−95.1	4.3	310.1	14.2	281
Z_1_	2018/04/21	90.9	54.8	163.1	9.5	230
Z_2_	2018/05/25	103.6	−31.8	181.9	14.7	274
Z_3_	2018/06/14	82.9	−26.8	193.1	10.4	261

Although water ice surface deposits are known to exist at mid‐latitudes (Carrozzo et al., 2009), in principle the surface temperatures pertaining to these observations are too high to trigger CO_2_ ice condensation (Table 3). Nevertheless, small CO_2_ ice patches are known to exist at mid‐low latitudes, in particular in steep craters' rims with pole‐facing slopes where shadows could preserve colder temperatures even during daytime (Vincendon, 2015; Vincendon et al., 2010). Another possible explanation for our detections is that residual night‐time frost (Khuller et al., 2021; Piqueux et al., 2016) is being observed for some time after sunrise. However, the local times of our detections concentrate within about 3–4 hr around noon, and the predicted surface temperatures are always higher than 190 K, way beyond the CO_2_ frost point. For the above reasons, we also investigate the possibility that our detections are related to atmospheric ice condensing at high altitude. LNO pixels' footprint is quite large (0.5 × 17.5 km^2^, Table 1) and, hence, makes it difficult to verify if these observations are related to small ice patches in mixed terrains (see also Sections 2.3 and 4.1) or to clouds. CO_2_ ice clouds are known to form in low temperature pockets at mesospheric altitudes, composed of populations of coarse grains as large as 7 μm (Clancy et al., 2019), as well as micron (about 1 μm) and sub‐micron (about 0.1 μm) particles (Aoki et al., 2018; Montmessin et al., 2006, 2007). The optical depth reported in literature is typically rather low (less than 0.5). On the other hand, frost grains expected size range varies from a few μm to some mm (Kieffer et al., 2000; Titus et al., 2001; Vincendon, 2015). Since the depth and shape of the 2.35 μm band are driven by the ice microphysics, a grain size analysis can be a useful way to test the interpretation of NOMAD mid‐latitude ice detections as either frost on the surface or CO_2_ ice clouds. For the sizes pertaining to the clouds regime, we perform radiative transfer simulations with the MITRA tool (Section 3.2), varying the optical thickness of the CO_2_ ice clouds in order to obtain a radiometric signal and a 2.35 μm absorption band that are comparable to those of the observed spectra. As we can see in Figure 8a, where the synthetic spectra are compared to the observation from zone Z_3_ in Table 3, all grain sizes in the range 0.1–7 μm do not yield any significant absorption at 2.35 μm (dashed orange, blue and cyan lines). The band depth increases for smaller grains, such as nanometer‐sized particles (order of magnitude 1–10 nm, dashed purple and solid green lines).

In this small‐size range, a good match with the observation in Figure 8a is obtained with a cloud with *r*_eff_ = 5 nm. However, a similar spectral shape can also be obtained with larger sizes pertaining to the surface ice regime (5 cm ice size, solid red line, obtained through the SNICAR tool, see Section 3.1). As reference, we verify that sizes this large (>1 cm) are needed in order to match the signal and the band shape of spectra related to the bulk of the South polar cap.

This dichotomy is verified in Figure 8b, showing the trend of the 2.35 μm band depth (that for simplicity we compute as 1−*R*_2.350_/*R*_2.352_, where *R* is the reflectance factor and the subscripts indicate the wavelength in microns) with very large variations of the CO_2_ ice effective radius. In this computation, we span from sizes comparable or smaller to those expected for CO_2_ ice clouds to sizes pertaining to the surface ice regime, up to slabs as large as 100 cm, always keeping constant the optical depth at *τ*_2_ = 0.4. From the figure it is evident how for sizes between approximately 100 nm and 1 mm the 2.35 μm band is practically absent. Instead, for both *r*_eff_ < 100 nm and *r*_eff_ > 1 mm the band depth increases. While the band depth increase for large sizes is ascribable to the increasing interaction of the light with the particle volume, the behavior for nanometric sizes is typical of the Rayleigh regime (i.e., when the size parameter 2πr/*λ* << 1, where *r* is the particle size and *λ* is the wavelength), where the absorption tends to progressively dominate the scattering with the decreasing sizes (Van De Hulst, 1981, chapter 6.13). Consistently with Figure 8a, two dimension regimes yield a band depth that is comparable to that of the observed spectra (order of magnitude 10% in all detections, red dashed line in Figure 8b), namely 1–10 nm and 1–10 cm.

As said above, the latter regime is consistent with extended and abundant surface ice deposits (Andrieu et al., 2018; Langevin et al., 2007) and, hence, pertains to particles larger than those expected for diurnal CO_2_ frost (about 1–10^3^ μm, Kieffer et al., 2000; Titus et al., 2001; Vincendon, 2015). For this reason, and taking into account the considered local times and surface temperatures (Table 3), we discuss if the size range 1–10 nm is preferable to describe the mid‐latitude observations. This regime is compatible with diurnal high altitude (about 100 km) CO_2_ ice clouds with grains smaller than 100 nm (Montmessin et al., 2006), higher in the atmosphere with respect to the expected larger ones (0.1–7 μm, Aoki et al., 2018; Clancy et al., 2019; Montmessin et al., 2007), and to which the 2.35 μm absorption band appears to be particularly sensitive, as demonstrated through our grains size investigation (Figure 8).

Under the clouds hypothesis, all detections B and D (Table 3) are seasonally compatible with CRISM (MY 29–33) and OMEGA (MY 28–30) CO_2_ ice clouds detections (Figure 14 in Clancy et al. [2019]; Figure 8 in Vincendon et al. [2011]) in the *L*_s_ ranges 40°–62° and 130°–135° respectively. In particular, detections D are also latitudinally consistent with MY27 detections from Montmessin et al. (2006) observed at *L*_s_ ∼135° and latitudes ∼−35° by the SPICAM instrument aboard Mars Express. Under the frost hypothesis, on the other hand, no matches of our detections have been found with the seasonal and spatial estimates from other authors (e.g., Vincendon, 2015; and Figure 3 in Vincendon et al. [2010]).

By performing the analysis described above to all detections in Table 3, we obtain the order of magnitude of the grains size and optical depth of the ice under the hypotheses that it is either pure frost on the surface or condensed in high altitude clouds. Taking into account the ice clouds hypothesis, we find grain sizes between 5 and 10 nm with optical depths in the range 0.1–0.3. Instead, under the hypothesis that those detections are related to surface CO_2_ frost, we find sizes in the range 1–10 cm with optical depths between 0.1 and 1.0.

Of course, the analysis described above cannot be considered as a quantitative retrieval of physical quantities but, as already stressed, provides an estimate of their order of magnitude. A more quantitative approach should rely on a number of assumptions that are still not well assessed, like the detailed instrumental response (e.g., AOTF and blaze functions, see Sections 2.2 and 3), the effect of non‐icy contaminants in the footprint, as well as uncertainties related to the spectral surface albedo or dust content. All these can easily affect the overall signal. For this reason, as a first stage we favored a qualitative approach, based on the statistical behavior of the spectral data set so far available, and trying to highlight that its information content about ice can be significant, although quite far from the main purposes which the instrument was devoted to.

Given the above discussion, we suggest that mid‐latitudes ice detections are more likely to be ascribed to clouds than to surface frost for context reasons and band depth estimates, resulting in the first detection of clouds through the study of the CO_2_ ice 2.35 μm absorption band. Nevertheless, a more conclusive discrimination between the two hypothesis is only achievable through a more comprehensive radiative transfer analysis of the 2.35 μm band as well as other spectral features, whose study is clearly beyond the scope of this paper and will be the subject of a future work (Section 6). Nevertheless, this analysis demonstrates that the 2.35 μm band, sampled by LNO order 189, has large potential to be exploited for the estimation of the grain size and optical depth of CO_2_ ice.

## 6 Conclusions and Future Work

In this work we explored NOMAD LNO information content of orders 169, 189, and 190 (Table 2) demonstrating that, although its main focus is on atmospheric gases, this data set can be exploited for the investigation of surface ice. Taking advantage of orders 169 and 190, partially covering the 2.7 μm band, we defined an index for the identification of abundant CO_2_ and H_2_O ices deposits (Ice Index, Section 4.1). We produced seasonal maps of the ice coverage that are in general in good agreement with MCD global climate model and with OMEGA data (Section 5), during both the peak and the sublimation phases of the polar caps. A seasonal map of the exposed CO_2_ ice was obtained using the 2.35 μm absorption band, which is well covered by LNO order 189 (Section 4.2). While the observed spectral features can only probe the first centimeters of ice deposits and hence cannot fully correlate with the total ice column predicted by global climate models, a meaningful comparison between observations and models has been achieved through an analysis of the average latitudinal extension of the polar caps and their seasonal variations. We verified that, while such an approach can be reliably used for the identification of abundant and spatially extended ice deposits, related to the inner crocus line (Section 5), it is not fully reliable in those transitional regions where icy to non‐icy terrains are strongly spatially mixed (taking also into account the relatively large NOMAD footprint).

We also investigated the impact of the 2018 global dust storm on the ice detection in the Southern polar cap during the spring season. In this case, the high concentration of dust in the atmosphere reduces the number of solar photons reaching the surface and thus back to the NOMAD detector, affecting the capability to spot the surface ice through the Ice Index investigation. On the other hand, we verified that the SAM algorithm is less affected by the presence of dust, making it a robust tool for the detection of spatially homogeneous and abundant CO_2_ ice deposits (Sections 4.3 and 5).

Through our analysis, CO_2_ ice detections were also found at mid‐equatorial latitudes, away from the polar regions. CO_2_ ice has already been detected at these latitudes both as surface ice/frost and high altitude clouds, and the discrimination of these conditions is not easy in nadir‐looking spectra.

While CO_2_ surface ice is known to exist at these latitudes in pole facing slopes remaining in shadow during daytime (Vincendon, 2015; Vincendon et al., 2010) or as residual night‐time frost in the early morning (Khuller et al., 2021; Piqueux et al., 2016), given the high temperatures and local times pertaining to these observations we also investigated the possibility that they are instead related to CO_2_ ice clouds. Through a grain size analysis (Section 5.1) we verified that two size regimes can reproduce the observations, namely 1–10 nm and 1–10 cm (see Section 5.1). The latter regime is too large for both the frost and the CO_2_ ice clouds hypotheses, while the 1–10 nm regime is compatible with fine grains in CO_2_ ice clouds condensing higher in the atmosphere (Montmessin et al., 2006) with respect to the larger dimension regimes of micron (1–7 μm) and sub‐micron (0.1 μm) particles. Given the above considerations, the clouds hypothesis results to be preferable and would imply the first detection of CO_2_ ice clouds through the 2.35 μm absorption band analysis. This investigation demonstrates how LNO order 189 has large potential to be exploited to retrieve CO_2_ ice microphysical properties. Moreover, this sets the basis for an in‐depth investigation of the band also in other datasets, like OMEGA and CRISM ones, even if characterized by a lower spectral resolution.

The analysis presented in this paper sheds some light on the possibility of conducting surface and aerosol science using a data set that is nominally conceived for the study of Martian trace gases, such that of NOMAD LNO, and opens the way for several follow up studies. An analysis of NOMAD UVIS channel information content, focused again on surface studies, is currently being performed as a continuation for the study presented in this paper. A dedicated quantitative radiative transfer analysis for the retrieval of surface ice microphysical and geometrical properties such as grains size, mass density and layers thickness, is already in progress and is the subject for a future paper. Moreover, an investigation focused on ice clouds detection is also being performed. Finally, an in depth radiative transfer analysis of the 2.35 μm feature in CO_2_ ice clouds spectra is planned.

## Data Availability

The OMEGA data used in this study are available from ESA's Planetary Science Archive at https://archives.esac.esa.int/psa/#!Table%20View/OMEGA=instrument. The Ice Index and SAM Similarity Index seasonal maps, the longitude‐latitude map of NOMAD LNO order 189 observations in Figure 2, and LNO order 189 spectra from Figure 5 (upper panel, blue line) and Figure 8 (panel a, black line) are made available through the Mendeley Data Repository at https://data.mendeley.com/datasets/cs72xnsz7k/2 (Oliva, 2022).
